# Prevalence and predictors of self-medication with antibiotics in Ethiopia: a systematic review and meta-analysis

**DOI:** 10.1186/s13756-024-01417-1

**Published:** 2024-06-09

**Authors:** Wondim Ayenew, Tewodros Ayalew Tessema, Yeniewa Kerie Anagaw, Ebrahim Abdela Siraj, Segenet Zewdie, Wudneh Simegn, Liknaw Workie Limenh, Chernet Tafere, Ashagrachew Tewabe Yayehrad

**Affiliations:** 1 Department of Social and Administrative Pharmacy, School of Pharmacy, College of Medicine and Health Sciences, University of Gondar, Gondar, Ethiopia; 2 Department of Pharmaceutics, School of Pharmacy, College of Medicine and Health Sciences, University of Gondar, Gondar, Ethiopia; 3 Department of Pharmaceutical Chemistry, School of Pharmacy, College of Medicine and Health Sciences, University of Gondar, Gondar, Ethiopia; 4 Department of Pharmaceutics, College of Medicine and Health Sciences, Bahir Dar University, Bahir Dar, Ethiopia; 5 Department of Social and Administrative Pharmacy, College of Medicine and Health Sciences, Injibara University, Injibara, Ethiopia

**Keywords:** Antibiotic self-medication, Associated factors, Prevalence, Ethiopia

## Abstract

**Introduction:**

Antibiotic self-medication is a global public health concern contributing to antibiotic resistance. This systematic review and meta-analysis aim to assess the prevalence of antibiotic self-medication and its associated factors in Ethiopia.

**Methods:**

A comprehensive search of electronic databases was conducted from MEDLINE (PubMed), Scopus, Google Scholar and Web of Science to identify relevant studies published between 2000 and 2024. Adult households, undergraduate university students and health care professionals who had taken antibiotics without a prescription in the household setting were included in this review. The primary outcome of this review is antibiotic self- medication. The random-effects model was used to estimate pooled prevalence rates. The outcome measure was analyzed with STATA version 17 software.

**Results:**

A total of nine studies were included in the Meta-analysis, comprising a sample size of 5908 participants. The pooled prevalence of antibiotic self-medication among Ethiopians was found to be 46.14 with 95% Confidence Interval [35.71, 56.57]. The most frequently used classes of self-medicated antibiotics were penicillins, followed by tetracyclines. Community pharmacies were the source of information that individuals utilized. The most common reported reasons for antibiotic self-medication include previous experience of treating a similar illness, to save cost, lack of time and avoiding waiting time for medical services. Participants having less than high school educational level was the most commonly reported factor associated with self-medication antibiotics.

**Conclusion:**

Antibiotic self-medication is a prevalent practice in Ethiopia. This underscores the need for targeted interventions such as educating people about the risks associated with using antibiotics without medical guidance, which results in a reduction in antibiotic resistance.

**Supplementary Information:**

The online version contains supplementary material available at 10.1186/s13756-024-01417-1.

## Background

The World Health Organization (WHO) describes self-medication as taking medications to address illnesses that someone has diagnosed without a doctor’s advice or supervision [1]. Self-medication with antibiotics in particular is a common practice worldwide [2]. In recent times, there has been consistent documentation of increasing rates of antibiotic self-medication worldwide [3]. Consequently, antibiotic self-medication has emerged as a major public health concern, garnering significant attention from researchers in the field of public health [4].

The use of antibiotics for self-identified illnesses without first seeing a trained healthcare provider is known as antibiotic self-medication [5]. This may result in the overuse of antibiotics, as well as other issues such as masking underlying symptoms, postponing or providing a false diagnosis, causing drug interactions, and hastening the development and dissemination of antibiotic resistance (1, 6–7). A larger portion of antibiotic misuse and self-medication is observed in developing countries [8]. Research indicates a higher prevalence of antibiotics misuse and self-medication in developing nations when compared to developed ones [9].

About 80% of antibiotics are thought to be utilized in communities outside recognized healthcare facilities in Low and Middle-Income Countries (LMIC), of which 20–50% are misused [10]. The waste of financial resources from extended treatment periods brought on by improper infection control and unpleasant effects are additional problems associated with antibiotic self-medication [5]. The increasing pandemic of antibiotic resistance has primarily affected Africa [11, 12]. More than half of the antibiotics used in communities, particularly in Africa, were reportedly sold without a prescription in 2011 [13].

Concern over Ethiopians self-medicating with antibiotics has grown recently [14]. Antibiotic self-medication was linked to easy availability of antibiotics without a prescription [15], a lack of knowledge regarding antibiotic resistance [16] and socioeconomic status [16, 17]. Antibiotic self-medication practices may also be influenced by poor healthcare infrastructure and restricted access to healthcare services in rural areas [18].

Ethiopia is known to have a significant burden of infectious diseases, including a high incidence of disease morbidity and mortality. This is likely because of increased rates of antimicrobial resistance (AMR). Additionally, there are indications that people, healthcare professionals, and society as a whole are using antibiotics excessively [19, 20]. The country has been putting numerous initiatives into practice to address the issue, including the responsible use of antibiotics, disease prevention and control, public surveillance suggesting the use of antibiotics, continuous guidelines, and enforcement. Nevertheless, a national study on the scope of antibiotic misuse was not conducted. Therefore, this review aimed to assess the prevalence and associated factors of antibiotic self-medication in Ethiopia.

## Methods

### Protocol and registration

The review protocol was developed and registered in International prospective register of systematic reviews with registration number CDR42023439111 and available at https://www.crd.york.ac.uk/prospero/#recordDetails. We followed the recommendation of PRISMA statement [21] to report this systematic review and meta-analysis [Supplementary file 1].

### Eligibility criteria

All published research on the prevalence of antibiotic self-medication in Ethiopia and its predictors was included in this systematic review. The study covered all cross-sectional observational quantitative studies that were published in English and carried out in households in Ethiopia. Dissertations and masters theses that have not been published were not included. Every study that wasn’t observational was disregarded. Qualitative or mixed method studies were excluded. Non-human studies and conference abstracts were also not included in the review.

### Information sources

Our research question focused on repeated database searches to find all the studies that met our inclusion criteria. In systematic reviews, it has been demonstrated that searching for multiple databases yields better results than searching for only one [22]. To find more research that might be included, the references of the identified studies were evaluated.

The inclusion rates of systematic reviews are increased when multiple databases are searched and references are verified [23]. From 2000 to 2024, pertinent research was looked for in the databases of MEDLINE (PubMed), Scopus, Google Scholar, and Web of Science. Additional possible resources, such as conference proceedings and books with abstracts, were also looked up.

### Search strategy

Using the PRISMA guidelines [21], an electronic systematic search was conducted on MEDLINE (PubMed), Scopus, Google Scholar, and Web of Science. Both index/subject terms and keywords were employed to expand the search approach. These phrases were combined using boolean operators (“OR,” “AND”) to create a search strategy. The search terms were “prevalence,” “proportion,” “magnitude,” “epidemiology,” “associated factors,” or “determinants,” as well as “antibiotic self-medication” or “self-prescription” or “Non-prescribed use of antibiotics” and “Ethiopia,” and the full syntax used for database search was ((((((((((‘Prevalence’[Mesh]) OR ‘proportion’ [Mesh]) OR ‘magnitude’ [Mesh]) OR ‘epidemiology’ [Mesh]) AND ‘associated factors’ [Mesh]) OR ‘determinants’ [Mesh]) AND ‘antibiotic self-medication’ [Mesh]) or ‘self-prescription’ [Mesh]) OR ‘Non-prescribed use of antibiotics’ [Mesh]) AND ‘Ethiopia’ [Mesh]) The Medical Subject Headings (MeSH) were employed in PubMed to align synonymous phrases. A preliminary scoping search was conducted on PROSPERO to make sure no previous review of a similar nature had been registered. The search was conducted from 01/05/2023 to 30/05/2023.

### Study selection

The review was designed by WA, AT, and EA. Independent assessors (WA, AT, EA, TA, YK, WS, and LW) select the study and extract the data. Titles and abstracts were independently evaluated by reviewers (WA, AT, EA, TA, YK, WS, and LW) and vetted against the qualifying criteria. Discrepancies were settled by SZ. The entire texts of the publications were retrieved for quality evaluation after the titles and/or abstracts were changed for potential inclusion.

### Data abstraction

The investigators created an Excel data extraction form. Subsequently, this form was used to extract and gather pertinent data. Authors’ names, publication years, regions, study designs, study settings, study participants, sampling methods, sample sizes, response rates, recall periods in months, prevalence (%) with 95% CI, factors associated with self-medication antibiotics, common antibiotics used in self-medication, source of antibiotics, perceived illnesses/symptoms for which antibiotics are used, and reasons for using antibiotics for self-medication are all included in the form. The review’s key outcome, or summary measure, is the prevalence of antibiotic self-medication and its determinants. The most common antibiotics used for self-medication, their source, the ailments or symptoms that people believe warrant their usage, and the motivation for their use were secondary outcomes of this review and meta-analysis.

### Assessment of the quality of included studies

The checklist for assessment of bias in systematic review of prevalence studies, created by Damian Hoy in 2012, was used to evaluate the characteristics of the included research. The Hoy checklist is the most popular method for evaluating bias in systematic reviews of prevalence studies. It is ten items total, split into two sections of the checklist. Six components evaluate internal validity (items 5 to 9 evaluate the domain of measurement bias, and item 10 evaluates bias related to the analysis). Four components (items 1–4) evaluate external validity (domains are selection and non-response bias). The total score of 0–4 was regarded as low quality, the total score 5–7 regarded as moderate quality and total score of 8–10 regarded as high quality [24].

### Data analysis

The data collected using the data abstraction format in Excel was exported to and analyzed using STATA version 17 statistical software. Data was presented quantitatively and in narrative form. DerSimonian-Laired random effect was performed to estimate the pooled prevalence of antibiotic self-medication in Ethiopia. Cochrane’s Q statistics, I^2^ and P values were used to check the heterogeneity of the studies. Meta regression analysis, subgroup and sensitivity analysis were performed in order to explain the cause of heterogeneity. The result was presented in a forest plot. The presence of publication bias was presented with a funnel plot.

## Results

**Fig. 1:**
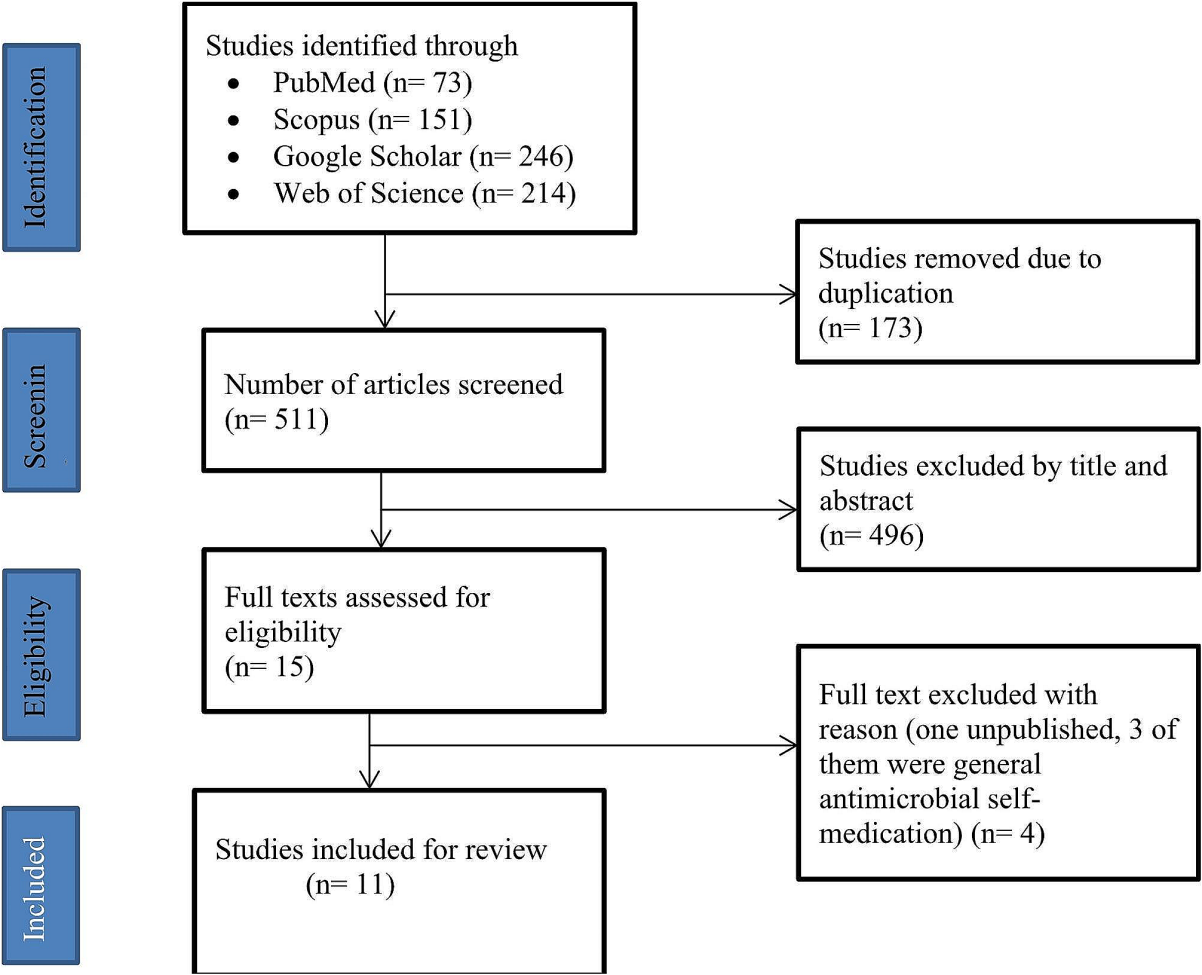
PRISMA flow diagram

About 73 articles were identified from PubMed, 151 from Scopus, 246 from Google Scholar, and 214 from Web of Science. 173 articles were duplicates and 511 articles were left for screening the titles and abstracts. About 496 articles were excluded. Then, 15 articles were left for further full text review. From these, 4 articles were excluded after reviewing of the full texts. Finally, 11 articles were eligible for the systematic review and meta-analysis (Fig. 1).

### Characteristics of included studies

All eleven studies selected for this review and meta-analysis were crossectional studies published in English from 2012 to 2024. About 5814 subjects were involved for the study. The samples were drawn using varied sampling methods from the general public (8/10 studies), undergraduate university students (1/10 studies), health professionals (physicians, pharmacists and nurses) (1/10 studies) from different settings such as households, university students, hospitals and drug retail outlets. The recall periods were varied among different studies, which range from 1 month to 12 months (Table 1 and 2).

**Table 1:** Characteristics of included studies

Author, year	Region	Study design	Study setting	Study participants	Sample size	Response rate	Recall period/in months/	Prevalence (%)	95% CI
Bogale et al., 2019 [25]	Addis Ababa	CS	Households	Adult people residing in the city	605	98.30%	6	67.30	63.6 − 71.0
Erku et al., 2017 [26]	Amhara	CS	Households	Adult communities in the town	720	90.30%	12	63.50	60.0–67.0
Eticha et al., 2014 [27]	Tigray	CS	Universities	Undergraduate Students	422	96.40%	3	44.50	39.8–49.2
Gebeyehu et al., 2015 [28]	Amhara	CS	Households	Adult communities in the town	1082	98.30%	2	18.00	15.7–20.3
Gebrekirstos et al., 2017 [29]	Tigray	CS	Drug retail outlet	Adult communities in the town	829	94%	2	47.10	43.7–50.5
Kassa et al., 2022 [30]	Addis Ababa	CS	Public hospitals	HCP(Physicians, pharmacists and nurses)	330	96%.	1	22.70	NR
Ayana et al., 2021 [31]	Oromia	CS	Drug retail outlet	Adult communities in the town	421	94.80%	1	43.100	38.6–48.1
Demissie et al., 2022 [32]	Oromia	CS	Households	Adult communities in the town	826	100%	12	38.90%	1.56–1.64
Simegn & Moges, 2022 [33]	Amhara	CS	Households	Adult communities in the town	421	96.70%	6	55.30%	50.6–60.2
Dache et al., 2021 [34]	SNNP	CS	Households	Adult communities in the town	582	97.60%	12	37.90%	34.0-41.5
Mossa et al., 2012 [35]	SNNP	CS	Households	Adult communities in the town	405	100%	12	27.30%	NR

CS: Crossectional study; HCP: Health care professionals; NR: Not reported

### Sampling method

**Table 2:** Sampling method used by included studies

Author, year	Sampling method
Bogale et al., 2019 [25]	Multistage sampling was used. Two subcities were randomly chosen. Then, four districts were proportionally selected from the chosen subcities using simple random sampling. Subsequently, 605 households were proportionally selected from the identified districts using systematic sampling methods based on a predetermined Kth value. Interviews were conducted with every Kth household, with the initial household chosen randomly. Throughout the data collection phase, house numbers served as the sampling frames. If a household declined participation, the next household in the sequence was approached. Interviews were conducted with heads of households until the final sample size was achieved. The study instruments were derived from a review of existing literature and previous studies.
Erku et al., 2017 [26]	The selection of households within administrative areas (kebeles) employed a multistage stratified random sampling technique. Initially, five administrative areas were randomly chosen to ensure a representative sample. The number of households to be interviewed in each administrative area was determined proportionally according to the total number of households in each kebele. Subsequently, a systematic random sampling method was utilized to select study participants within these areas. In cases where more than one eligible respondent was found within a selected household, a respondent was chosen through a lottery method. The questionnaire used in the study was developed by modifying items from a previously employed instrument. The items were meticulously reviewed for relevance by a team of experts, including experienced clinical pharmacists and public health professionals.
Eticha et al., 2014 [27]	The selection of departments involved a stratified sampling technique. Three departments were selected, and then further stratified based on study year. Respondents from each study year were selected proportionally according to their population size, utilizing simple random sampling techniques. The structured questionnaire was developed through a comprehensive review of relevant literature and previously standardized instruments.
Gebeyehu et al., 2015 [28]	The sampling methodology involved a multistage stratified random sampling approach to select households in both rural and urban kebeles. To ensure representation, three urban and three rural kebeles were randomly selected using a simple random sampling technique. Within these selected kebeles, a systematic random sampling technique was employed to choose study participants. Data collection utilized a pre-tested and structured questionnaire to gather relevant information.
Gebrekirstos et al., 2017 [29]	Samples districts were chosen using a simple random sampling technique. Drug retail outlets samples were selected randomly. Proportional to sample size technique was used to determine the number of outlets selected. Subsequently, study participants were recruited using consecutive sampling technique. A pre-test and structured questionnaire was employed to gather relevant data.
Kassa et al., 2022 [30]	The study employed a multi-stage sampling technique. Samples were selected using a lottery method. The sampling frame was taken from human resource department. The number of HCPs to be included from each hospital was determined proportionally based on the size of the staff. Following this, HCPs were stratified into physicians, nurses, and pharmacy professionals, and the final sample size was allocated proportionally according to the respective number of HCPs from each department in each hospital. Convenient sampling method was then utilized to select the final sampling units. The data collection tool was prepared after reviewing previous studies on the same issue.
Ayana et al., 2021 [31]	A simple random sampling technique was utilized to samples. Subsequently, the history of antibiotic purchasers was assessed for each selected pharmacy and drug store. Based on this historical data, systematic random sampling methods were employed to select study participants from each establishment. Every third purchaser was selected based on their sequence of visiting the drug retail outlet, with the initial study subject determined randomly through a lottery method. The sample size was allocated proportionally to each pharmacy or drug store. The questionnaire used for data collection was prepared from previous studies. Exit interviews were conducted immediately after a person purchased antibiotics, with their consent, at the pharmacies or drug stores.
Demissie et al., 2022 [32]	The selection of sample households relied on the frame comprising three kebeles, along with their respective household numbers, obtained from the kebele administration offices. Proportional probability to size sampling was employed to allocate a proportional sample size for each kebele. Subsequently, a systematic sampling technique was applied, determining the interval by dividing the total number of households in in the study area by the final sample size. Then, every Kth household whose members voluntarily participated was interviewed based on their sequence of house numbers. In cases where two or more eligible respondents were present in the same household, only one of them was randomly selected and included in the study. Face-to-face interviews were conducted with eligible respondents using structured, pre-tested questionnaires in selected households where occupants were available during data collection.
Simegn & Moges, 2022 [33]	The study employed a stratified sampling technique to proportionally allocate households to each kebele administration. Within the city, five kebeles and three rural kebeles were selected using a lottery method. The number of households was obtained from the City administration, and lists of households with their respective addresses were acquired from each kebele administrative office. From each stratum, samples were drawn proportionally to their size using the number of households as the sampling frame. Household selection within urban and rural kebeles was conducted using a systematic random sampling technique. The sampling interval for each kebele was determined by dividing the total number of households by its proportionally allocated sample size. Subsequently, every Kth household was interviewed, with the first household selected through a lottery method. Data collection utilized a semi-structured, pretested questionnaire adapted from previous studies. Interviews were conducted with the head of the household or a member designated as the next head or responsible person of the household.
Dache et al., 2021 [34]	A multistage sampling approach was utilized to categorize study subjects. Initially, four kebeles were randomly selected from the six kebeles in the town. Subsequently, the number of households to be selected from each chosen kebele was proportionally allocated based on the total number of households in that kebele. Lists of households were obtained from each kebele health post, and a sampling frame was independently developed for each selected kebele based on census results. The calculated sample size was then proportionally allocated to all selected kebeles based on their respective number of eligible households. Study subjects were selected using a simple random sampling procedure. A door-to-door interview approach was employed for each kebele to identify suitable study participants until the requested sample size was attained. In instances where there were two or more eligible households, a chance method was used to select one of them. The questionnaire used for data collection was prepared by modifying related literature to ensure alignment with the study objectives and conceptual framework. Data were collected using interviewer-administered structured and pretested questionnaires.
Mossa et al., 2012 [35]	The sampling procedure involved randomly selecting a sample of adult individuals from the town using a multi-stage stratified clustered sampling technique. Initially, residential areas within the town were randomly selected. From these areas, sample households were randomly chosen. Subsequently, one individual from each household was interviewed. To collect information, a structured and pre-tested questionnaire was utilized. The validity of the questionnaire was assessed through in-depth discussions.

### Quality assessment of included studies

Eleven studies were assessed for risk of bias. All studies showed a low-level risk of bias (Table 3).

#### External validity

1. Was the study’s target population a close representation of the national population in relation to relevant variables?
2. Was the sampling frame a true or close representation of the target population?
3. Was some form of random selection used to select the sample, OR was a census undertaken?
4. Was the likelihood of nonresponse bias minimal?

#### Internal validity

5. Were data collected directly from the subjects (as opposed to a proxy)?
6. Was an acceptable case definition used in the study?
7. Was the study instrument that measured the parameter of interest shown to have validity and reliability?
8. Was the same mode of data collection used for all subjects?
9. Was the length of the shortest prevalence period for the parameter of interest appropriate?
10. Were the numerator(s) and denominator(s) for the parameter of interest appropriate?

**Table 3:** Quality assessment of included studies

Author, year	1	2	3	4	5	6	7	8	9	10	Total score
Bogale et al., 2019 [25]	0	1	1	1	1	1	0	1	1	1	8
Erku et al., 2017 [26]	0	1	1	1	1	1	0	1	1	1	8
Eticha et al., 2014 [27]	0	1	1	1	1	1	0	1	1	1	8
Gebeyehu et al., 2015 [28]	0	1	1	1	1	1	0	1	1	1	8
Gebrekirstos et al., 2017 [29]	0	1	1	1	1	1	0	1	1	1	8
Kassa et al., 2022 [30]	0	1	1	1	1	1	0	1	0	1	7
Ayana et al., 2021 [31]	0	1	1	1	1	1	0	1	0	1	7
Demissie et al., 2022 [32]	0	1	1	1	1	1	0	1	1	1	8
Simegn & Moges, 2022 [33]	0	1	1	1	1	1	0	1	1	1	8
Dache et al., 2021 [34]	0	1	1	1	1	1	0	1	1	1	8
Mossa et al., 2012 [35]	0	1	1	1	1	1	0	1	1	1	8

### Prevalence of antibiotic self-medication

**Fig. 2:**
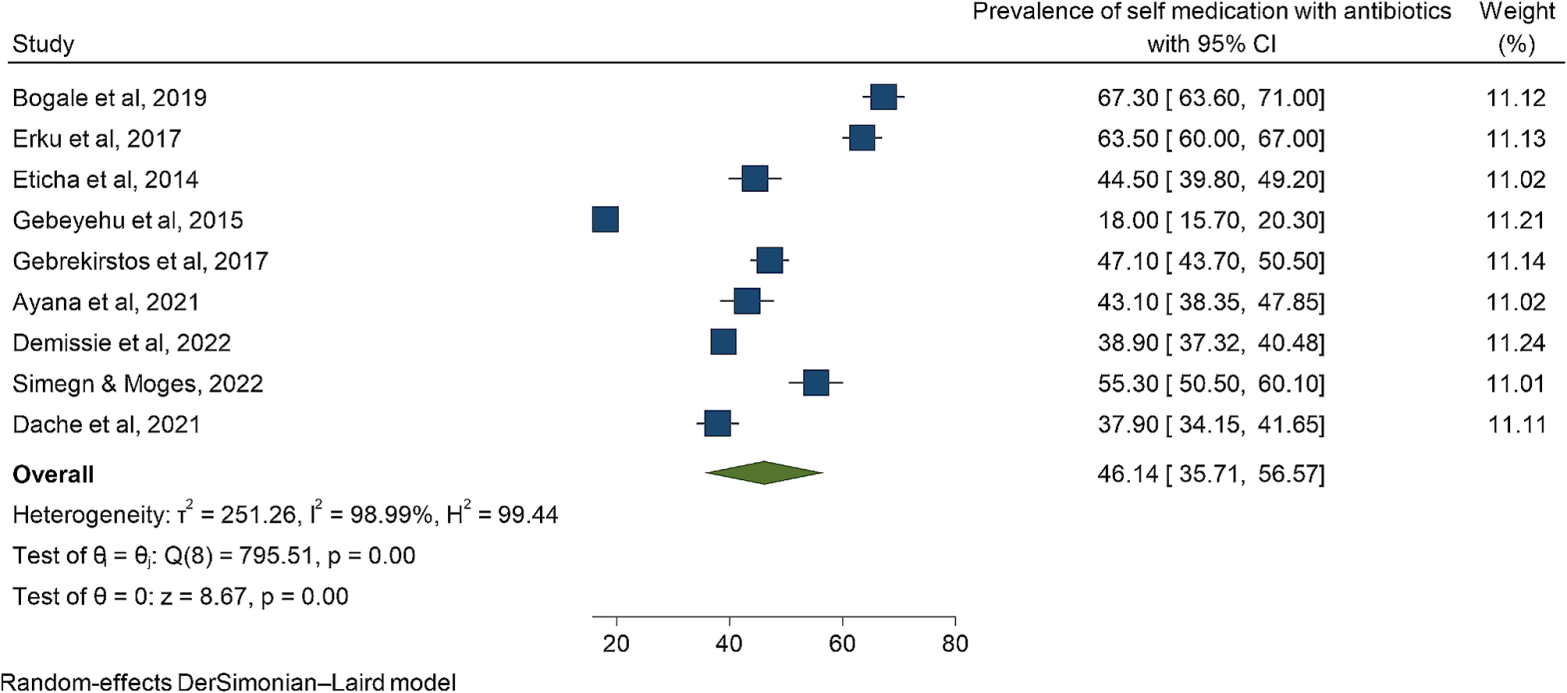
A summary of forest plot that showed the overall pooled prevalence of antibiotic self-medication in Ethiopia

Nine studies were eligible for meta-analysis. The overall prevalence of antibiotic self-medication in this study is 46.14 [35.71, 56.57]. The prevalence varied across regions which ranged from 18.0% to 0 67.3% (Fig. 2).

### Subgroup and Meta regression analysis

The selected studies exhibited significant heterogeneity (I^2^ = 98.99%). This suggests that the inconsistency among studies was greater than what would occur randomly, resulting in an inconsistent overall estimate of the proportion of antibiotic self-medication. This was taken into account while estimating the over prevalence of antibiotic self-medication using a random effect model. Meta regression analysis was evaluated along with a subgroup analysis in order to explain the cause of heterogeneity. Sample size and response rate were used in the Meta regression analysis and none of them were significant and did not explain the source of heterogeneity (Table 4).

**Table 4:** Meta regression analysis of the studies based on sample size and response rate

meta_es	Coefficient	Std. err	Z	*P*>|z|	[95% conf. interval]	
Sample size	− 0.0290937	0.0209821	-1.39	0.166	− 0.0702178	0.0120304
Response rate	-191.8384	165.2825	-1.16	0.246	-515.7861	132.1093
_cons	249.9722	157.8263	1.58	0.113	-59.36161	559.3061

Test of residual homogeneity: Q_res = chi2 [6] = 337.70 Prob > Q_res = 0.0000

Subgroup analyzes were carried out based on region and study setting. The analysis showed that the pooled prevalence of antibiotic self-medication is almost similar to the pooled prevalence in Amhara, Tigray and Oromia regions, where studies in Addis Ababa and Sidama are higher and lower than the pooled prevalence of antibiotic self-medication respectively.

**Fig. 3:**
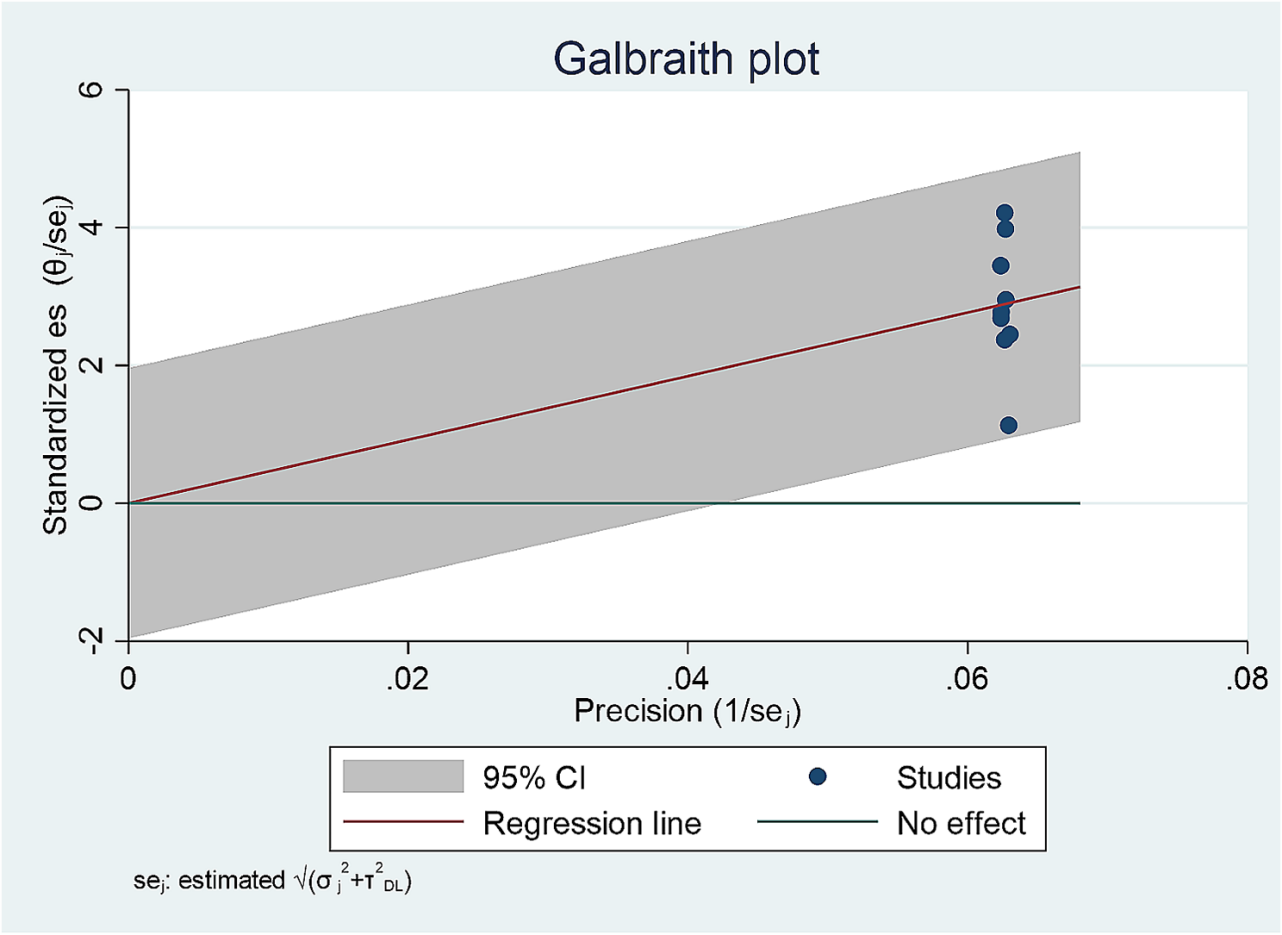
Galbraith plot

Subgroup analysis based on a study setting showed that the pooled prevalence of antibiotic self-medication in household, university and pharmacy retail is similar to the pooled prevalence of antibiotic self-medication. However, heterogeneity obviously not decreased. In addition, a Galbraith plot was drawn to identify some studies that were obviously different from others. But the Galbraith plot showed the absence of substantial heterogeneity since all studies lie within the 95% CI region (shaded area) (Fig. 3).

### Sensitivity analysis

**Fig. 4:**
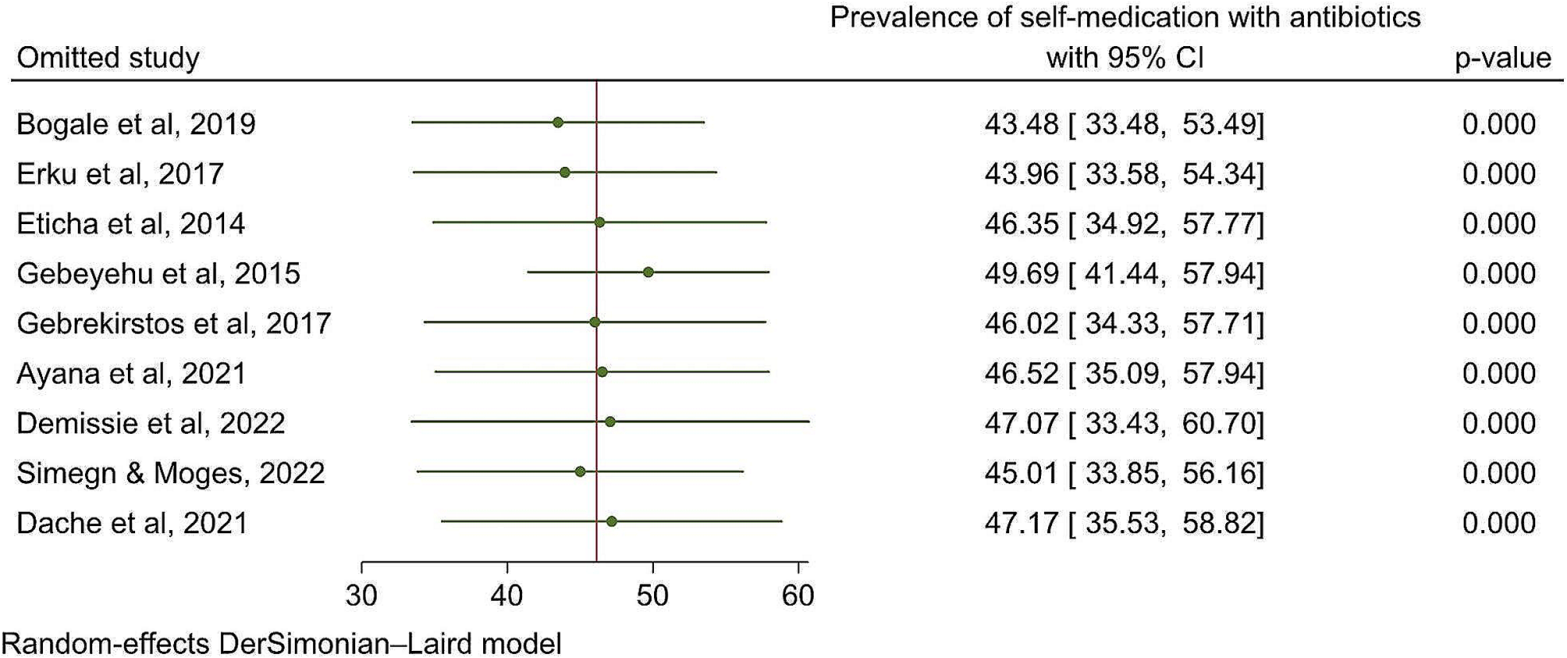
Leave-one-out sensitivity analysis

To investigate the impact of each individual study on the pooled prevalence of antibiotic self-medication, a leave-one-out meta-analysis was conducted. When each study was removed from the analysis, the pooled estimate prevalence of antibiotic self-medication fell between the confidence interval of the pooled estimated prevalence of antibiotic self-medication, indicating that no single study could affect the statistically significant difference (Fig. 4).

### Publication bias

**Fig. 5:**
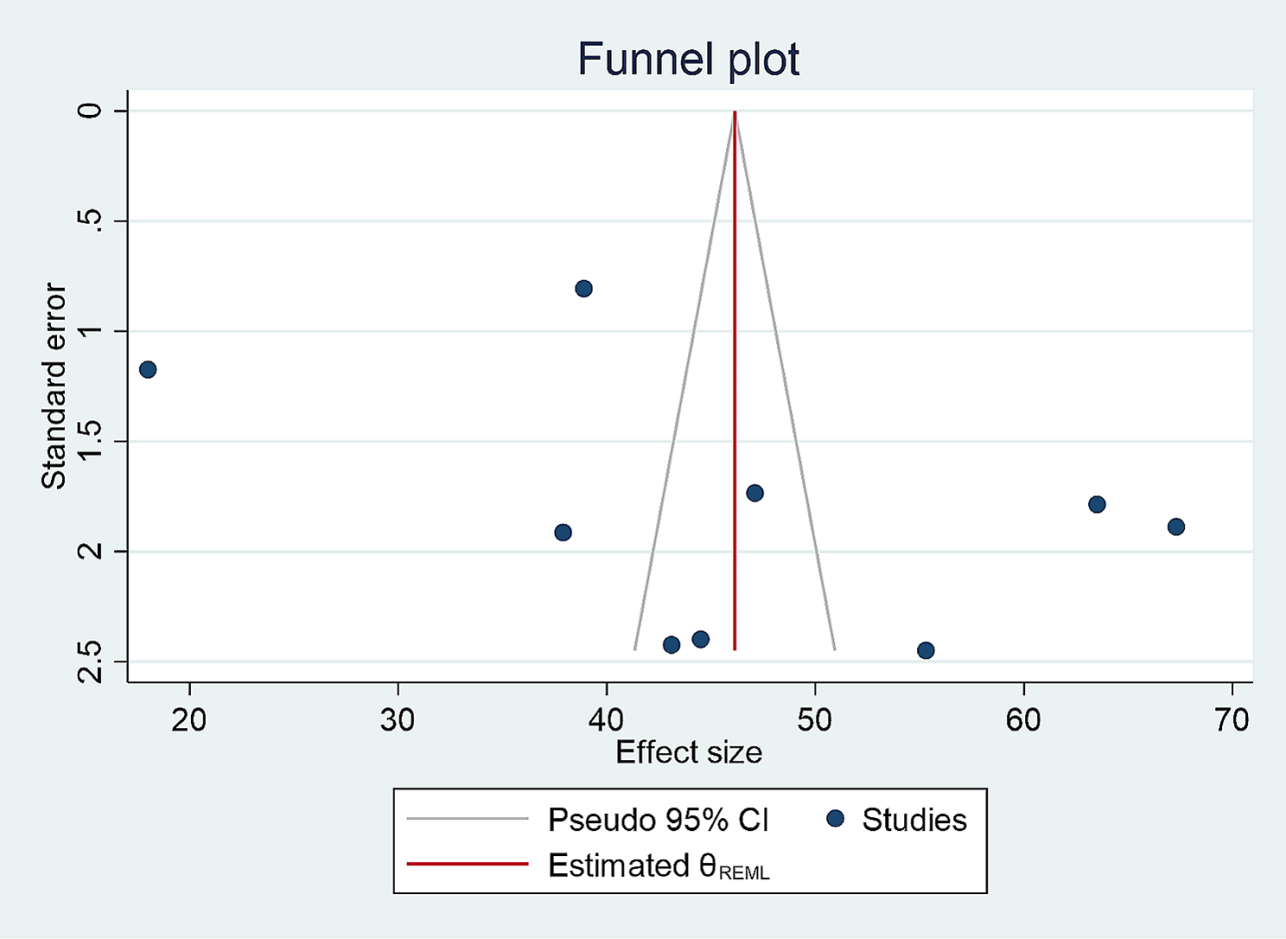
Funnel plot

Nonparametric trim-and-fill analysis of publication bias was performed using funnel plot to confirm the evidence of publication bias. Despite the asymmetry of the funnel plot, Egger and Begg’s tests revealed that publication bias was not statistically significant (P values of 0.2621 and 0.3481 respectively) (Fig. 5).

### Common antibiotics used for self-medication

Eight different classes of antibiotics were self-medicated by study participants and the antibiotics commonly used in self-medication include penicillins (10 studies), tetracyclines (6 studies), fluoroquinolones (5 studies), Cephalosporin (2 studies), sulphonamides (1 study), macrolides (1 study), Chloramphenicol (1 study) and aminoglycosides (1 study). The most frequently used classes of self-medicated antibiotics were penicillins followed by tetracyclines (Table 5).

**Table 5:** Common antibiotics used for self-medication

Author, year	Classes of antibiotics	Antibiotics	Percentage used
Bogale et al. 2019 [25]		Penicillins	67.2%
		Fluoroquinolones	23%
		Sulphonamides	40%
Erku et al. 2017 [26]		Penicillins	72%
		Tetracyclines	19%
		Fluoroquinolones	8.9%
Eticha et al. 2014 [27]		Penicillins	51.7%
		Fluoroquinolones	12.9%
		Tetracyclines	5.5%
Gebeyehu et al. 2015 [28]		Penicillins	75.5%
		Fluoroquinolones	7.2%
		Tetracyclines	10.6%
Kassa et al. 2022 [30]		Penicillins,	41.6%
		fluoroquinolones,	40.3%
		Sulphonamides	8%
		Cephalosporin	6%
		others	4%
Ayana et al. 2021 [31]	Penicillins	Amoxicillin	43.6
	Penicillins	Ampicillin	11.6
	Sulphonamides	Cotrimoxazole	8.7
	Macrolides	azithromycin	7.6
	Penicillins	Cloxacillin	4.1
	Penicillins	Augumentin	3.5
	Cephalosporin	Cefalexin	2.9
	Tetracyclines	Tetracycline	2.3
	Fluoroquinolones	Ciprofloxacin	1.7
	Macrolides	Erythromycin	1.2
	Amphenicols	Chloramphenicol	1.2
	aminoglycosides	Gentamicin	0.6
	Cephalosporin	cefixime	0.6
	Penicillins	Phenoxymethylpenicillin	0.6
Demissie et al. 2022 [32]	Penicillins	amoxicillin	22.1%
	Fluoroquinolones	ciprofloxacin	9.7%
	Tetracyclines and Fluoroquinolones	doxycycline and ciprofloxacin	8.7%
	Sulphonamides	co-trimoxazole	6.2%
Simegn & Moges, 2022 [33]	Penicillins	Amoxicillin	45%
	Fluoroquinolones	Ciprofloxacin	36%
	Penicillins	Amoxicillin with clavulanic acid	24%
Dache et al. 2021 [34]	Penicillins	Amoxicillin	53.4
	Tetracyclines	Doxycycline	5.6
	Penicillins	Cloxacillin	5.2
		Others	6
Mossa et al. 2012 [35]	Penicillins	Amoxicillin	13.5
	Penicillins	Ampicillin	5.0
	Tetracyclines	Tetracycline	6.8
	Fluoroquinolones	Norfloxacin	3.3
	Fluoroquinolones	Ciprofloxacin	8.5
	Tetracyclines	Doxycycline	5.0
		Others	3.3

### Source of antibiotics

Studies reported that participants were obtained information from various sources for antibiotics used in self-medication in Ethiopia. These include community pharmacies (8/10 studies), family/ relatives/ friends/neighbors (7/10 studies), leftovers from previous treatment(4/10 studies), patent medicine stores (3/10 studies), hospital pharmacies (2/10 studies), health workers such as doctors, nurse (2/10 studies), private health facilities (1/10 studies), sample from medical representatives (1/10 study), by sharing with the others (1/10 study), and kiosks (1/10 study) (Table 6).

**Table 6:** Source of antibiotics

Author, year	Source of antibiotics	Percentage
Bogale et al. 2019 [25]	Community pharmacies	82.3
	Patent medicine stores	2.0
	Private health facilities	11.0
	Public hospital pharmacies	3.1
Erku et al. 2017 [26]	Community pharmacies	36.8
	Health workers	44.1
	Family/friends	19.1
Eticha et al. 2014 [27]	Community pharmacies	83.0
	Patent medicine store	58.9
	Friends/family	29.5
	Leftovers from previous treatment	28.6
Gebeyehu et al. 2015 [28]	Community pharmacies	15.5
	Friends/relatives	15.7
Kassa et al. 2022 [30]	hospital pharmacies	51.0
	Community pharmacies	39.0
	Leftovers from previously used antibiotics	7.0
	sample from medical representatives	1.0
	family/friends/neighbors	2.0
Demissie et al. 2022 [32]	medicine dispensers	70.8
	previous doctor’s prescription	25.6
	friends	3.6
Simegn & Moges, 2022 [33]	Retail outlet pharmacy	41.8
	From friends or family members	28.7
	By sharing with the others	15.0
Dache et al. 2021 [34]	doctor or nurse	62.1
Mossa et al. 2012 [35]	Neighbors	5.1
	Left over past prescribe	7.7
	Kiosks	17.9
	Pharmacy	59.0
	Other	10.2

### Perceived illnesses/symptoms for which antibiotics are used for self-medication

Four studies reported the perceived illnesses/symptoms for which antibiotics were used for self-medication by study participants. The common indications reported for use for antibiotic self-medication include upper respiratory tract infection (URTI), gastro intestinal problems, common febrile illness, body aches, skin problems, urinary tract problems (Table 7).

**Table 7:** Perceived illnesses/symptoms for which antibiotics are used for self-medication

Author, year	Perceived illnesses/symptoms	Percentage
Bogale et al. 2019 [25]	Upper respiratory tract infection (URTI).	60.1%
	Common cold	(32.6%),
	common febrile illness	(28.7%),
	diarrhea	(27.1%),
	malaria	(3.7%).
Kassa et al. 2022 [30]	Respiratory problems	29 40.3
	Gastro intestinal problems	28 38.9
	Skin problems	3 4.2
	Urinary tract problems	6 8.3
	Unidentified cases	6 8.3
Demissie et al. 2022 [32]	Aches and pains	(15.5%)
	Typhoid and typhus	(8.1%)
	Cough	(6.3%)
	Community acquired Pneumonia (CAP)	(3.4%)
	Diarrhea	(4.3%)
	amebiasis	(2.5%)
	Tonsillitis	(1.7%)
	wound	(1.5)
Mossa et al. 2012 [35]	Headache	30(38.5)
	Fever	28(35.9)
	Cough	11(14.1)
	Diarrhea	8(10.2)
	Abdominal pain	8(10.2)
	Joint & back pain	28(35.9)
	Nausea & vomiting	6(8.5)
	Other	6(8.5)

#### Reason for which antibiotics are used for self-medication

Seven studies reported the reason for which antibiotics were used for self-medication. The most common reported reasons of antibiotic self-medication include previous experience, to save cost, lack of time and avoiding waiting time (Table 8).

**Table 8:** Reason for which antibiotics are used for self-medication

Author, year	Reason for which antibiotics	Percentage
Bogale et al. 2019 [25]	previous experience for the same symptom	60.3%
Eticha et al. 2014 [27]	Prior experience of treating a similar illness	78 69.6
	Minor illness	49 43.8
	Avoiding waiting time for medical services	41 36.6
	Cost-effectiveness	36 32.1
	Others	24 21.4
Kassa et al. 2022 [30]	Being familiar with treatment	31 (43.1%)
	need for quick relief	25 (34.7%
	lack of time	14%
	easy access to medicines	12%
	to save cost	10%
	to maintain privacy	3%
Ayana et al. 2021 [31]	save time	62.2%
	save money	111 (64.5%)
	to get well soon	111 (64.5%)
	previous experience with the same disease	64.0%
Demissie et al. 2022 [32]	prolonged waiting to get service in health institutions	(39.9%)
	Medical treatment of the previous similar symptoms	(19.8%)
	lack of time to visit health institutions	(16.4%)
Dache et al. 2021 [34]	Long delays in health facility	99 46.1
	Cost-cutting	13 6
	Busy day’s program	90 41.8
	Previous experience of the same symptoms	13 (6%)
Mossa et al. 2012 [35]	Low-cost alternative	6 (7.7)
	Disease is minor	15 (19.2)
	Avoiding waiting time for medical services	16 (20.5)
	No time	10 (12.8)
	Distance of health facility	7 (9.0)
	Emergency case	13 (16.7)
	Other	11 (14.1)

### Factors associated with self-medication antibiotics

All of the studies reported the associated factors with antibiotics self-medication despite differences in factors across studies. Low educational level, age (18–34 years) and gender i.e. being male were common significantly associated factors reported and considered as factors for antibiotic self-medication practice in Ethiopia. Low educational level was the most commonly reported factor associated with self-medication antibiotics (Table 9).

**Table 9:** Factors associated with antibiotic self-medication

Author, year	Variables	AOR
Bogale et al., 2019 [25]	Age 18–30	8.45 (2.55, 27.96)
	No education	6.39 (1.45, 28.19)
	Low income	2.55 (1.18, 5.50)
Erku et al., 2017 [26]	Low educational status	5.01 (2.62, 9.34)
	Employed	2.12, (1.81, 7.29)
	Unsatisfied with healthcare services provided	5.41 (2.71, 14.21)
Eticha et al., 2014 [27]	Protestant religion	2.26 (1.19, 4.27)
Gebeyehu et al., 2015 [28]	< 25 years	4.45 (1.54, 12.85)
	25–34 years	2.73 (1.03, 7.24)
	Poor educational status	4.21 (1.47, 12.07)
	Engaged with a regular job	1.94 (1.13, 3.32)
	Unsatisfied with healthcare services	3.51 (2.14, 5.78)
Kassa et al., 2022 [30]	None of the socio-demographic factors tested in multivariable logistic regression were found to be associated with HCPs self-medication	
Ayana et al., 2021 [31]	Being male	2.21 (1.276, 3.835)
	residing in rural area	3.659 (1.479, 9.054)
	holding diploma	0.120 (0.025, 0.591)
	hold BSC degree	0.050 (0.007, 0.378)
	being farmer	0.034 (0.004, 0.285)
Demissie et al., 2022 [32]	Being male	1.53 (0.489, 0.869)
	no health insurance scheme	2.16 (0.274, 0.779)
	availability of some drugs in shop	12.98 (0.017, 0.353)
Simegn & Moges, 2022 [33]	Educational level (8–10 grade)	4.10 (1.28, 13.12)
	using mass media as a source of information	2.23 (1.24, 4.27)
	relying on previous experience for source of information	2.02 (1.23, 3.31)
	having awareness of antibiotics resistance	2.45 (1.34, 4.50)
	good knowledge of antimicrobial resistance	1.81 (1.11, 2.97)
Dache et al., 2021 [34]	Being employed (adjusted	3.45 (1.98, 6.02)
	age 25–34 years	2.89 (1.43, 5.84)
	being male	1.90 (1.20, 3.02)
	seeking modern healthcare in private clinic	2.54 (1.20, 5.36)
	delayed waiting time in healthcare facilities	4.87 (2.17, 10.91)
	experienced with similar symptom/disease	3.02 (1.89, 4.83)
	family size above five	8.92 (3.56, 22.38)
Mossa et al., 2012 [35]	Level of monthly income and educational status significantly influence pattern of antibiotics and antimalarial self-medication (*P* < 0.05)	

## Discussion

The prevalence of antibiotic self-medication is a concern in Ethiopia based on the meta-analysis, indicating a high overall rate of 46.14%. The use of antibiotics without a prescription occurs despite their prescription being only legal status in most countries [13]. This self-medication use of antibiotics contributes to accelerating the emergence and spread of antimicrobial resistance (AMR) [1, 36, 37].

Variations across regions from 18.0 to 67.3% suggest differing cultural or healthcare factors influencing this behavior. Numerous studies corroborate this trend. For instance, the prevalence of antibiotic self-medication in Iran was found 53.3% [38], 20–25% in Europe [34], 48.8% in Africa [39]. These discrepancies could be attributed to variations in healthcare accessibility, education, regulatory policies, and cultural beliefs regarding antibiotics. It underscores the global significance of addressing this issue to combat antibiotic resistance.

The overall pooled prevalence in our study is found to be higher than that reported in systematic reviews from South East Asia [40] and the WHO Eastern Mediterranean Region [41]. Poor regulation of antibiotic sales resulting from the absence of policies or laxity in law enforcement makes antibiotics easily available for self-medication [13].

The classes of antibiotics most commonly self-medicated by study participants were penicillin followed by tetracyclines. It is consistent with other systematic reviews reported by the Middle East [42] and Europe [43]. It also aligns with the general knowledge that penicillins are widely used due to their efficacy against a broad range of infections, while tetracyclines are often chosen for their effectiveness against various bacterial illnesses. The varying use across different antibiotic classes might reflect regional availability, familiarity, or perceived effectiveness by users.

Multiple studies have also reported similar trends in the classes of antibiotics used in self-medication. For instance, a study in Saudi Arabia [44] found penicillins to be commonly self-medicated, consistent with our data. Another study in Nigeria [45] observed tetracyclines were among the most frequently self-administered antibiotics. Additionally, the WHO report on antibiotic use highlighted the widespread misuse of penicillins and tetracyclines globally. These studies collectively echo the prevalence of penicillins and tetracyclines in self-medication practices, suggesting a recurring pattern across various regions in the choice of these antibiotic classes (47).

In our study, the most common sources for antibiotics used in self-medication in Ethiopia were community pharmacies. Studies conducted in the Euro-Mediterranean region and developing countries have been reported that pharmacists were the main source of information for SMA (41, 48).

Several studies worldwide have also highlighted similar sources for obtaining antibiotics for self-medication. For instance, a study in Nigeria found community pharmacies and friends/relatives as common sources for self-medicated antibiotics [45]. Moreover, a study in Palestine noted community pharmacies and leftover medications from previous treatments among the primary sources for self-medication with antibiotics (49). Similarly, a study across various European countries identified community pharmacies and obtaining antibiotics from acquaintances as frequent sources for self-medication [34].

These studies emphasizing the role of community pharmacies in facilitating antibiotic self-medication practice, which could contribute to antibiotic misuse and resistance. This shows that community pharmacists are responsible for the extensive antibiotic misuse in the community. Therefore, the laws and regulations the country has should be strongly implemented in the community pharmacies. Because lax regulations or enforcement might allow pharmacies to dispense antibiotics without proper prescriptions, contributing to their frequent use as sources for self-medication.

The reported indications for antibiotic self-medication in this study align with commonly perceived illnesses/symptoms worldwide (Upper Respiratory Tract Infections, Gastrointestinal Problems, Febrile Illnesses, Body Aches, Skin Problems and Urinary Tract Problems). Studies conducted globally corroborate these findings and suggest a consistent pattern where individuals tend to self-medicate with antibiotics for similar perceived illnesses/symptoms across different regions, emphasizing the need for targeted education on appropriate antibiotic use [13, 16, 34, 40].

The reasons behind antibiotic self-medication, including previous experience, cost-saving, time constraints, and avoiding waiting times, align with findings from various studies conducted globally in Iran [38], Saudi Arabia [44], Nigeria [45] and across European countries [34], and Palestine (49). These reasons are recurrent across different regions, indicating common motivations for individuals resorting to self-medication with antibiotics, underscoring the need for improved access to healthcare and education on appropriate antibiotic use.

In the current study, low educational level, age (18–34 years) and gender i.e. being male were, significantly, the most common reported factors for antibiotic self-medication practice in Ethiopia. Low educational level was the most commonly reported factor associated with self-medication antibiotics. This shows the need for promoting literacy among communities and sensitization of the public as a vital strategy to also reduce antibiotic self-medication. Illiteracy is a driver of antibiotic self-medication as individuals and entire communities have less opportunity to be aware of the health risks associated with antibiotic self-medication (50). Special attention should be given to educating the public and healthcare providers on drugs used for self-medication and their impact on the development of antimicrobial resistance should be provided by the community.

This review and meta-analysis has certain limitations. Studies have been concentrated in certain regions, limiting the generalizability of findings to the entire country. Variations in study methodologies and populations could introduce heterogeneity affecting the pooled prevalence.

## Conclusions

Antibiotic self-medication is a substantial issue in Ethiopia, with almost half the population engaging in this practice. A prevalence rate of 46.14% indicates a significant public health concern. It is considered high when compared to similar studies conducted in other countries or regions. The World Health Organization (WHO) discourages self-medication with antibiotics due to the risks associated with incorrect usage, such as antibiotic resistance. Any prevalence rate above zero indicates a potential concern, but a rate of 46.14% is particularly high relative to the WHO’s recommendation. Penicillins and tetracyclines were frequently self-medicated. Community pharmacies were a major source, and reasons included past experiences, cost-saving, lack of time, and avoiding waiting times. Lower education levels were the major determinant of antibiotic self-medication.

### Recommendations

A targeted interventions such as educating people about the risks associated with using antibiotics without medical guidance which results in reduction in antibiotic resistance is needed. This review and meta-analysis exhibited significant clinical heterogeneity among the studies included, thus it should be considered with caution “Abbreviations.

AMR: Antimicrobial resistance; EFDA: Ethiopian Food and Drug Authority; LMIC: Low and Middle-Income Countries; PRISMA: Preferred Reporting Items for Systematic Review and Meta-Analysis; WHO: World Health Organization.

### Electronic supplementary material

Below is the link to the electronic supplementary material.

Supplementary Material 1

## Data Availability

All data generated or analyzed are included in this review.
